# Optimization of Thermoelectric Performance of Ag_2_Te Films via a Co-Sputtering Method

**DOI:** 10.3390/nano14211762

**Published:** 2024-11-03

**Authors:** Hanwen Xu, Zhongzhao Zha, Fu Li, Guangxing Liang, Jingting Luo, Zhuanghao Zheng, Yue-Xing Chen

**Affiliations:** Institute of Thin Film Physics and Applications, Shenzhen Key Laboratory of Advanced Thin Films and Applications, Key Laboratory of Optoelectronic Devices and Systems of Ministry of Education and Guangdong Province, State Key Laboratory of Radio Frequency Heterogeneous Integration, College of Physics and Optoelectronic Engineering, Shenzhen University, Shenzhen 518060, China; 2200451025@email.szu.edu.cn (H.X.); 2300451029@email.szu.edu.cn (Z.Z.); lifu@szu.edu.cn (F.L.); lgx@szu.edu.cn (G.L.); luojt@szu.edu.cn (J.L.); zhengzh@szu.edu.cn (Z.Z.)

**Keywords:** thermoelectric, Ag_2_Te, thin films, electrical transport properties

## Abstract

Providing self-powered energy for wearable electronic devices is currently an important research direction in the field of thermoelectric (TE) thin films. In this study, a simple dual-source magnetron sputtering method was used to prepare Ag_2_Te thin films, which exhibit good TE properties at room temperature, and the growth temperature and subsequent annealing process were optimized to obtain high-quality films. The experimental results show that films grown at a substrate temperature of 280 °C exhibit a high power factor (PF) of ~3.95 μW/cm·K^2^ at room temperature, which is further improved to 4.79 μW/cm·K^2^ after optimal annealing treatment, and a highest PF of ~7.85 μW/cm·K^2^ was observed at 200 °C. Appropriate annealing temperature effectively increases the carrier mobility of the Ag_2_Te films and adjusts the Ag/Te ratio to make the composition closer to the stoichiometric ratio, thus promoting the enhancement of electrical transport properties. A TE device with five legs was assembled using as-fabricated Ag_2_Te thin films. With a temperature difference of 40 K, the device was able to generate an output voltage of approximately 14.43 mV and a corresponding power of about 50.52 nW. This work not only prepared a high-performance Ag_2_Te film but also demonstrated its application prospects in the field of self-powered electronic devices.

## 1. Introduction

Semiconductor thermoelectric devices are convenient to manufacture, reliable to operate, and do not require moving parts or fluid media. They can become an indispensable and important technology in the current military and civilian fields such as power generation and refrigeration [1,2,3,4,5,6,7]. The thermoelectric conversion efficiency of thermoelectric devices is mainly determined by the dimensionless figure of merit *ZT*, which is defined as *ZT* = *S*^2^*σT*/*κ*, where *S* is the Seebeck coefficient, *σ* is the conductivity, *T* is the absolute temperature, and *κ* is the thermal conductivity. The portion of the formula related to electrical properties is referred to as the power factor (PF), defined as *PF* = *S*^2^*σ* [8,9,10,11,12].

In recent years, with the widespread application and expanding market of wearable electronic devices, providing long-lasting and lightweight power sources for these devices has become an important potential direction for the application of thermoelectric materials. Although traditional batteries can provide power to devices, their limited lifespan, charging requirements, and potential environmental hazards have spurred the exploration of alternative energy sources [13]. In this context, flexible thermoelectric films that can directly convert body heat or ambient temperature differences into electrical energy have become a promising alternative [14]. Flexible thermoelectric thin films offer advantages such as being lightweight and easy to integrate, allowing them to be applied in various complex device structures [15]. Particularly in wearable electronic products, they can harness small temperature differences from the body or environment to continuously generate electricity, thereby reducing the reliance on traditional batteries [16]. The development of high-performance flexible thermoelectric films represents a significant area of research interest within this field [17].

The current research on flexible thermoelectric thin films is primarily focused on enhancing the conventional thermoelectric Bi_2_Te_3_-based system [18]. This is due to the fact that this system material exhibits the most favorable thermoelectric properties and has been the subject of extensive study [19]. However, the Bi_2_Te_2_ system has certain disadvantages, including limitations in high-temperature thermoelectric properties, poor thermal stability, and high cost [20]. Additionally, there are other material systems with narrow band gaps, including Ag_2_Se (~0.07 eV) [21], Ag_2_Te (~0.1–0.5 eV) [22], and Cu_2_Se (~0.8–1.4 eV) [23], which have also been the subjects of research and have yielded some results.

Ag_2_Te exhibits excellent electrical conductivity and has the potential to become an excellent thermoelectric material, which has attracted widespread attention. Ag_2_Te has three structural forms: α, β, and γ [24]. The low-temperature monoclinic phase α-Ag_2_Te is stable below 150 °C, and the low-temperature monoclinic phase α-Ag_2_Te transforms into the high-temperature face-centered cubic phase β-Ag_2_Te (stable between 150 °C and 800 °C), while γ-Ag_2_Te exists stably in the range of 800 °C~960 °C [25]. At room temperature, the film with a monoclinic phase has high electron mobility (~6000 cm^2^ V^−1^S^−1^) and low thermal conductivity (~1 Wm^−1^K^−1^) [26]. Hence, α-Ag_2_Te is expected to be a kind of thermoelectric material with excellent properties in low-temperature regions [27]. For example, Masaki et al. [28] studied the preparation of polycrystalline sintered samples of Ag_2_Te and measured the electrical properties from room temperature to 627 °C. The maximum power factor of 5.99 μW/cm·K^2^ at 67 °C was achieved. As for thin films, Meng et al. [29] synthesized Ag-rich Ag_2_Te nanowires by wet chemistry and prepared flexible PVP/Ag/Ag_2_Te ternary composite films on nylon membranes by vacuum-assisted filtration and heat treatment. For the Ag/Te composite films with an initial molar ratio of 6:1, the power factor as high as 2.165 μW/cm·K^2^ was obtained at 27 °C and increased to 3.701 μW/cm·K^2^ at 120 °C. Amish et al. [30] prepared a flexible Ag₂Te-nylon composite film by vacuum filtration. The film exhibited a high Seebeck coefficient (−135.5 μV/K) and low thermal conductivity (0.29 W/mK), resulting in an improved power factor of about 4.5 μW/cm·K^2^ and the *ZT* of about 0.48 at 40 °C. Zhou et al. [31] fabricated a high-strength, highly flexible n-type thermoelectric film based on Ag_2_Te nano-barrels/poly (vinylidene fluoride) by a solution-processable method. The power rating of the film exceeds 0.3 μW/cm·K^2^ at room temperature. These works provided variable effective ways to prepare Ag_2_Te films with high TE performance and good flexibility for portable TE generators. However, the thermoelectric performance of the currently reported work is still lower than that of the traditional Bi_2_Te_3_-based system [32] and the newly developed Ag_2_Se-based system [33], and there is still much room for improvement in its electrical transport performance. Hence, it is necessary to prepare higher thermoelectric performance Ag_2_Te-based films through appropriate methods. In this work, we perform a simple dual-source magnetron sputtering method to prepare Ag_2_Te thin films. The effects of growth temperature and annealing process on the thermoelectric properties were investigated. As a result, an improved power factor of ~4.79 μW/cm·K^2^ at room temperature and a highest PF of ~7.85 μW/cm·K^2^ at 200 °C were achieved. A TE device with five legs was also assembled by using as-fabricated Ag_2_Te thin films. The output voltage of 14.43 mV and a corresponding output power of 50.52 nW demonstrate the potential of its application in the field of wearable electronic devices.

## 2. Experimental

**Figure 1:**
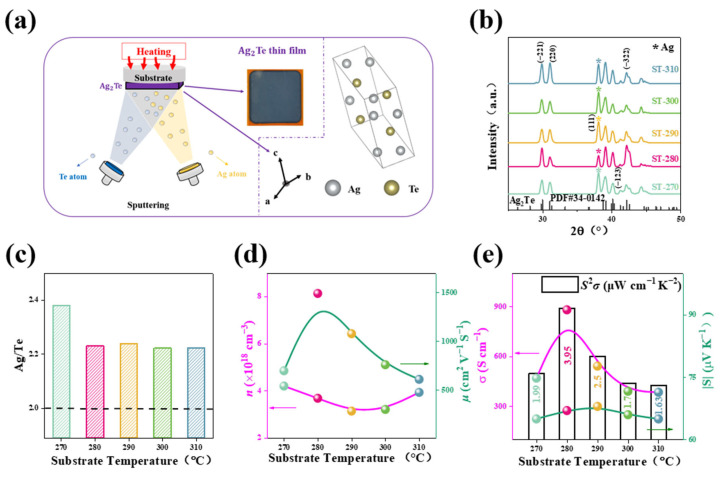
(**a**) Schematic diagram of the preparation process of Ag_2_Te thin films; inset figures show the optical photographs of Ag_2_Te thin films and the crystal structure of Ag_2_Te. (**b**) XRD patterns of samples at different substrate temperatures. (**c**) Ag/Te ratio of films as a function of substrate temperatures. (**d**) Room temperature electrical conductivity (*σ*), Seebeck coefficient (*S*), power factor (*PF*). (**e**) Carrier concentration and mobility of Ag_2_Te thin films prepared at different substrate temperatures.

Ag_2_Te thin films were synthesized using a dual-source co-sputtering method (Figure 1a), employing Ag (99%) and Te (99%) targets. The effects of substrate temperature and annealing temperature on the thermoelectric transport properties of the films were investigated. A flexible polyimide (PI) substrate with a thickness of 0.125 mm and 70% transmittance was selected [34]. Firstly, the PI substrates were ultrasonically cleaned in ultrapure water, acetone, and anhydrous ethanol for 15 min, respectively [35]. Then, the PI substrates were dried using a nitrogen blow gun. The substrate was placed on the sample holder of a vacuum magnetron sputtering machine, where it was preheated to 270 °C, 280 °C, 290 °C, 300 °C, and 310 °C to study the influence of substrate temperature on the thermoelectric properties of the films. For the sputtering process, the power of the Ag and Te targets was kept at 25 W, the vacuum pressure was maintained at 8 × 10^−4^ Pa, and the working pressure was 0.5 Pa [36]. The co-sputtering deposition time was set to 5 min. After deposition, the sample was naturally cooled to room temperature in the vacuum chamber. Subsequently, the sample was transferred to a heating stage inside a glove box, where it was annealed for 30 min at temperatures of 150 °C, 200 °C, 250 °C, 300 °C, and 350 °C to explore the impact of annealing temperature on the thermoelectric properties of the thin films.

Subsequently, we simply prepared a thermoelectric device. The optimized five Ag_2_Te films (2.5 mm × 18 mm) were deposited on a flexible substrate. Subsequently, to reduce contact resistance, a layer of silver film was plated at both ends of each strip as electrodes. Finally, these five strips were connected in series to assemble the Ag_2_Te film device [37]. Using a simple heating device to create a temperature difference between the device and the external environment, the short-circuit current, open-circuit voltage, and output power of the film device were measured using a Keithley 2400 source meter (Keithley Instruments, Cleveland, OH, USA) and its Ke2400 program.

The crystal structure of the films was characterized using an X-ray diffractometer (XRD.D/max 2500, Rigaku Corporation, Tokyo, Japan, using CuKα radiation) scanning from 10°to 70° at a rate of 10° per minute. The valence states were assessed using X-ray photoelectron spectroscopy (XPS, Thermo Escalab 250XI, Thermo Fisher Scientific, Waltham, MA, USA). The surface morphology and chemical composition of the films were examined using scanning electron microscopy (SEM, Zeiss-spra 55, Oberkochen, Baden-Württemberg, Germany) and energy dispersive spectrometry (SEM-EDS, Bruker Quantax 200, Billerica, MA, USA). The thickness of the film was measured by a profilometer (Dektak XT, BRUKER, Ettlingen, Germany). Furthermore, the Hall performance (n and μ) at room temperature was measured using the HL5500PC Nano Metrics Hall measurement system. The in-plane conductivity (σ) and Seebeck coefficient (S) were recorded with the SBA458 instrument from Nezsch, Selb, Germany.

## 3. Results and Discussion

As shown in Figure 1a, the Ag_2_Te thin films were prepared using magnetron sputtering technology, and the optical image of Ag_2_Te displays a dark blue–violet film. During the preparation process, the substrate temperature was controlled to adjust the microstructure and electrical properties of the Ag_2_Te thin films. The crystal structure of the films grows along different crystallographic axes and is characterized by X-ray diffraction (XRD) to determine the crystal structure. Figure 1b shows the X-ray diffraction (XRD) patterns of Ag_2_Te thin films at different substrate temperatures, labeled as ST-270, ST-280, ST-290, ST-300, and ST-310, corresponding to substrate temperatures of 270 °C, 280 °C, 290 °C, 300 °C, and 310 °C, respectively. The diffraction peaks of Ag_2_Te thin films at different substrate temperatures mainly appear on the (−221), (220), and (−322) crystal planes. In the XRD patterns, a strong Ag (111) diffraction peak appears, which is due to the presence of elemental silver. The EDS composition of the samples at different substrate temperatures was measured (Figure 1c), and it can be seen that The Ag/Te ratio in all the films exceeds 2.0, further confirming the presence of Ag observed in the XRD results. This finding is also consistent with reports in the literature. At a substrate temperature of 270 °C, the Ag/Te ratio in the Ag_2_Te films reaches its highest value, approximately 2.4. After this point, as the temperature increases, the ratio gradually stabilizes. This indicates that while the substrate temperature has some influence on the composition of Ag_2_Te, at higher temperatures, the stoichiometry of the films remains around 2.2.

Figure 1d shows the variations in electrical conductivity (*σ*) and Seebeck coefficient (*S*) of the Ag_2_Te thin films. As the substrate temperature increases, the electrical conductivity reaches a peak at 280 °C, about 900 S/cm. However, the absolute value of *S* (|*S*|) also reaches its maximum at the same temperature, but then sharply decreases at higher temperatures. This is because the increase in electrical conductivity is usually associated with an increase in carrier mobility, while the Seebeck coefficient tends to decrease as the carrier concentration increases. The power factor (*S*^2^*σ*) reaches its maximum value (3.95 μW/cm·K^2^) at 280 °C, indicating that the thermoelectric performance of the film is optimal at this temperature. Figure 1e reveals the trends of carrier concentration (n) and mobility (μ) with changing substrate temperature. It can be observed that the carrier concentration decreases first and then increases, reaching its minimum at around 290 °C, approximately 3.14 × 10^18^ cm^−3^. This supports the observed changes in the Seebeck coefficient at different substrate temperatures. The mobility reaches its maximum at around 280 °C, approximately 1500 cm^2^/V·s. This supports the observed changes in *σ* and *S* at different substrate temperatures. This is because the increase in electrical conductivity is usually associated with the increase in carrier mobility, while the Seebeck coefficient tends to decrease as the carrier concentration increases [38]. On the other hand, the presence of silver in the material limits its overall thermoelectric performance. Although silver can enhance certain properties, it also introduces scattering mechanisms that hinder charge carrier mobility, ultimately reducing the effectiveness of the thermoelectric conversion. Hence, the thermoelectric performance of Ag_2_Te thin films is highly sensitive to substrate temperature. Figure S1a–c shows variable temperature data for film *σ* and *S* and *PF* for Ag_2_Te films. The ST-280 sample exhibited better conductivity throughout the test temperature, which majorly resulted in its higher power factor. Therefore, the most appropriate substrate temperature process parameters are selected. However, although the Ag_2_Te films prepared at 280 °C achieved a power factor of 3.95 μW/cm·K^2^ at room temperature, there is still much room for improvement in their performance. Therefore, further annealing treatment will be applied to continue improving the thermoelectric efficiency of the thin film material.

**Figure 2:**
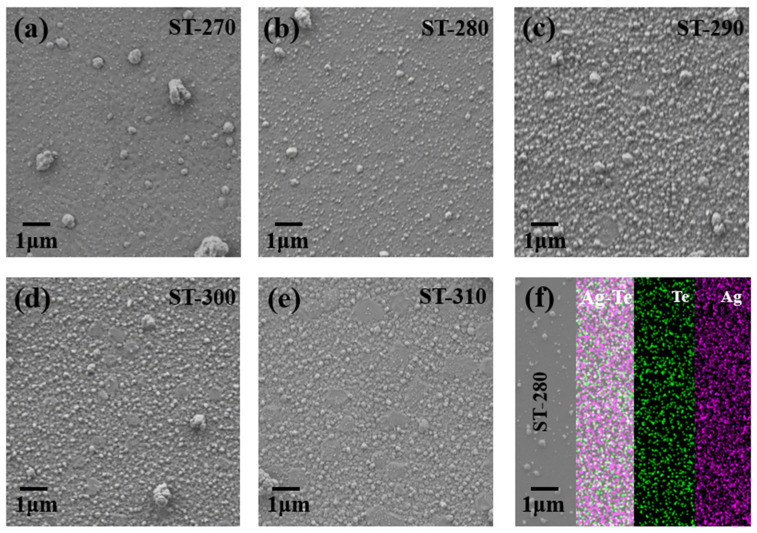
(**a**–**e**) Surface morphology of Ag_2_Te films prepared at different substrate temperatures and (**f**) elemental distribution analysis for ST-280 sample.

Figure 2a–e shows that the temperature of the substrate exerts a considerable influence on the properties of Ag_2_Te. As the temperature rises, the film surface becomes increasingly homogeneous, and the grains are smaller and more evenly distributed, indicating enhanced crystallinity. This is due to the gradual decrease in the Ag content as the temperature rises. It can be observed that the surface is characterized by the presence of grains of varying sizes and numbers, with the grains becoming increasingly smaller as the temperature rises. The results obtained from the EDS analysis and element mappings for Ag and Se indicate the existence of Ag (Figure S2), which is consistent with the XRD results and the findings reported in the literature. We further performed the elemental distribution analysis for the ST-280 sample, which shows a uniform distribution of Ag and Te elements (Figure 2f), indicating that the main phase of Ag_2_Te and the film was deposited in high quality. Based on the undeveloped thermoelectric properties and the existence of impurity Ag phases, we believe that it is necessary to adopt further optimization strategies to modify the composition and enhance the thermoelectric performance of Ag_2_Te films.

**Figure 3:**
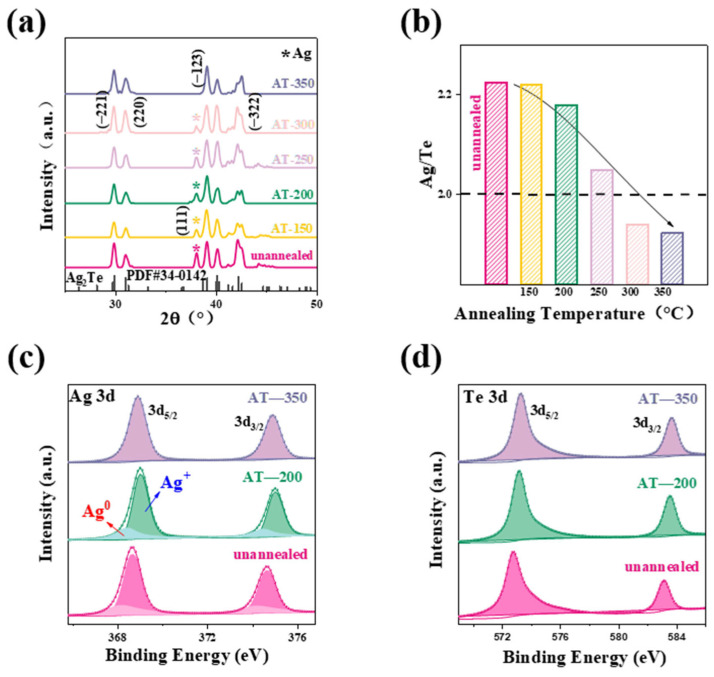
(**a**) XRD patterns of annealed samples at different temperatures and of the unannealed sample. (**b**) Ag/Te ratio of films as a function of annealing temperatures and unannealed. (**c**,**d**) X-ray photoelectron spectroscopy (XPS) of Ag 3d and Te 3d.

Hence, to further improve the thermoelectric performance of Ag_2_Te, an annealing treatment process is executed by transferring the deposited Ag_2_Te (ST-280), which obtained the highest *PF* (~3.95 μW/cm·K^2^) at room temperature in the previous step, to a heating stage inside a glove box The influence of annealing temperature on the thermoelectric performance and composited is investigated. For the convenience of description, in this section, ST280 will be labeled as the unannealed sample. Figure 3a shows the XRD patterns of Ag_2_Te thin films that are unannealed and annealed at different temperatures (150 °C, 200 °C, 250 °C, 300 °C, and 350 °C). All annealed samples maintained the Ag_2_Te main phase well. As the annealing temperature increases, the Ag (111) diffraction peak gradually weakens, indicating the reduction of silver particles. This result is further confirmed by the decrease of the Ag/Te ratio as the annealing temperature increases (Figure 3b). Notably, at temperatures above 300 °C, the Ag/Te ratio falls below 2.0, indicating that the Te element might start to volatilize at higher temperatures. The decrease of Ag suggests that the Ag_2_Te is close to the stoichiometric ratio with a single phase, and it is favorable for optimizing the electrical transport properties. Figure 3c shows the variation in the chemical state of Ag before and after annealing treatment. The oxidation state changes of Ag can be analyzed through the Ag 3d spectrum in the XPS results. In the unannealed sample, Ag is primarily present in the metallic state (Ag^0^), as shown by the narrower Ag_3d_ peak, which may indicate incomplete incorporation of Ag in the film. After annealing, the peak corresponding to the oxidation state of Ag* is significantly enhanced, indicating that the chemical bond between Ag and Te is enhanced during the annealing process, and Ag changes from a metallic state to a compound state. This result is consistent with the changes in XRD and Ag/Te ratio (Figure 3a,b). Furthermore, the annealing process helps stabilize the oxidation state of Ag in the film, and the chemical state of Ag tends to be stable at higher temperatures. Figure 3d shows the chemical state of Te in the unannealed and annealed samples. At 350 °C, the intensity of the Te peak does not increase further, indicating that the chemical state of Te tends to be stable at high temperatures. XPS analysis of Ag and Te shows that the chemical states of Ag* and Te^2−^ are more stable after annealing at 200 °C, indicating that this temperature effectively improves the chemical stability of the Ag_2_Te film. The XRD plots showed that all the annealed samples retained the dominant phase of Ag_2_Te, whereas the diffraction peaks of silver (Ag) weakened as the temperature increased, suggesting that there was a decrease in the free silver and a subsequent decrease in the Ag/Te ratio. When the annealing temperature is above 300 °C, Te may start to volatilize and Ag_2_Te approaches the stoichiometric ratio, thus contributing to the enhancement of electrical transport properties. On the other hand, XPS analysis shows that Ag exists mainly in the metallic state in the unannealed sample, while the oxidation state of Ag⁺ is significantly enhanced after annealing, indicating that the chemical bonding between Ag and Te is strengthened during the annealing process. Meanwhile, the chemical states of Ag and Te tend to be stable at high temperatures. This process ultimately contributes to the improvement of the thermoelectric properties of the film, allowing it to remain reliable under different environmental conditions.

**Figure 4:**
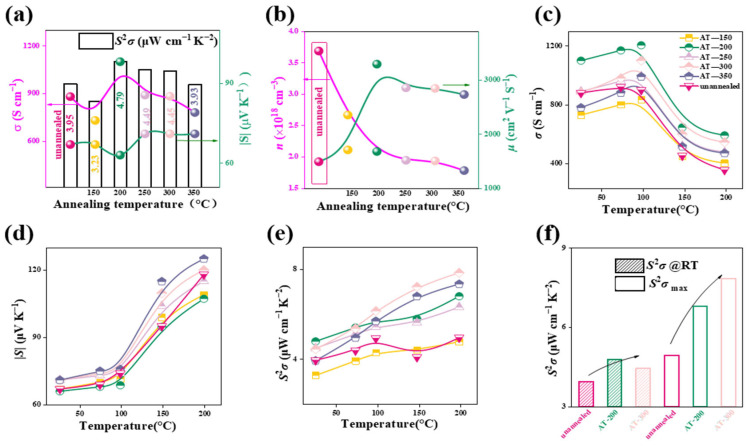
(**a**) Room temperature electrical conductivity (*σ*), Seebeck coefficient (*S*), power factor (*PF*), (**b**) carrier concentration and mobility of Ag_2_Te thin films prepared at different annealing temperatures and unannealed. (**c**–**e**) Temperature-dependent electrical conductivity (*σ*), Seebeck coefficient (*S*), and power factor (*PF*) at different annealing temperatures and of the unannealed sample. (**f**) Trend of *PF* value changes for different samples at room temperature and the maximum *PF* value at varying temperatures.

Figure 4a shows the variation in electrical transport properties as a function of annealing temperature at room temperature for unannealed and annealed samples. The electrical conductivity of the unannealed sample is about 880 S/cm, which reaches its peak for AT-200, approximately 1100 S/cm, and then gradually decreases at higher annealing temperatures. On the other hand, the absolute Seebeck coefficient remains relatively stable at around 70 μV/K for all Ag_2_Te films, contributing to the similar variation tendency with that of conductivity, and achieves the highest value 4.79 μW/cm·K^2^ obtained at an annealing temperature of 200 degrees (AT-200). This value is 21% higher than that of the unannealed film, confirming the improvement effect of annealing on thermoelectric performance.

To explore the effect of annealing treatment on electrical transport properties, the *n* of the film was measured and the *μ* was estimated with the formula *μ* = *σ*/*en* [39]. The results are plotted in Figure 4b. As the annealing temperature increases, the *n* gradually decreases, reaching its lowest value at 350 °C, around 1.8 × 10^18^ cm^−3^. The reduction is majorly due to the decline in the Ag/Te ratio (Figure 3b) after annealing treatment. The reduction of Ag leading to changes in carrier concentration is reasonable because Ag, as a metallic element, can contribute a large number of free electrons to participate in conduction. In addition, Ag does not contribute much to the *S*, which can also explain the small fluctuation of the *S* in Figure 4a. On the other hand, the *μ* shows an opposite trend to the *n*, and its value is greatly improved by the annealing process. It reaches a maximum value of ~3291 cm^2^/V·s for the AT-200 sample, which supports its highest *σ*. Combined with the greatly reduced *n*, the *σ* of the Ag_2_Te film after annealing has not been significantly improved. However, the annealing treatment helps reduce grain boundary scattering and regulate Ag/Te to stoichiometric ratios, resulting in improved carrier mobility. This conclusion is further supported by Figure S3a–e, which demonstrates a discernible increase in grain size as the temperature rises. Concurrently, the number of Ag particles within the material gradually decreases and ultimately vanishes at elevated temperatures.

Figure 4c,d illustrates the variation of electrical conductivity and Seebeck coefficient with temperature. Significant changes are observed around 150 °C, due to the phase transition of low-temperature monoclinic α-Ag_2_Te to high-temperature face-centered cubic β-Ag_2_Te. Figure 4e shows how the power factor changes with different measurement temperatures. As the temperature increases, the power factor gradually increases, and samples annealed at 200 °C–300 °C exhibit higher power factors at elevated temperatures. Notably, the sample annealed at 300 °C shows the highest PF value of 7.85 μW/cm·K^2^ at 200 °C. This indicates that proper annealing temperature can effectively enhance the thermoelectric performance of the film, especially under high-temperature operating conditions.

Figure 4f compares the room temperature and maximum power factors. It can be seen that the annealed samples exhibit improved power factors at both room temperature and higher temperatures, with the sample annealed at 200 °C showing significant improvements in both room temperature and maximum power factors. The sample annealed at 300 °C exhibits the highest power factor at elevated temperatures. This suggests that annealing can improve the thermoelectric performance of the material at both room temperature and high temperatures, making it suitable for a wider range of operating conditions.

**Figure 5:**
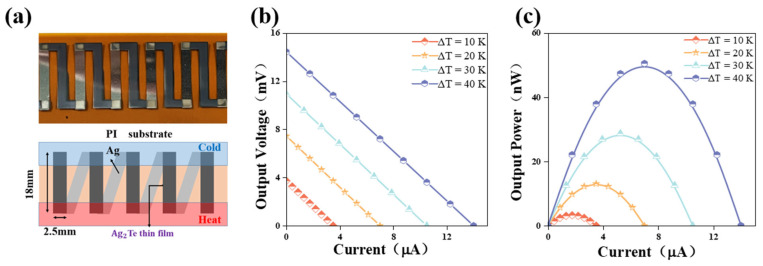
(**a**) The schematic diagram (**bottom** image) and the optical photo of Ag_2_Te TE thin film generator (**upper**). (**b**) The *V*_out_ and (**c**) output *P*_out_ depends on *I* with a Δ*T* ranging from 10 K to 40 K.

To demonstrate the application potential of Ag_2_Te films, we assembled a flexible device consisting of five Ag_2_Te legs, as shown in Figure 5a. The hot end and cold end are located at the lower and upper parts of the device, respectively, and the voltage signal is obtained by setting the temperature differences (Δ*T*) at both ends. The schematic of the device shows the dimensions of each leg, which is 2.5 mm wide and 18 mm long. Each Ag_2_Te leg is connected by an Ag electrode. As a result, the open voltage *V*_out_ as a function of current (I) is shown in Figure 5b when Δ*T* ranges from 10 K to 40 K. When 40 K Δ*T* is applied, the output voltage and current reach approximately 14.43 mV and 14 *μ*A, respectively. Correspondingly, as shown in Figure 5c, when Δ*T* is 40 K, the maximum output power is about 50.52 nW. This design demonstrates the potential of flexible thermoelectric devices based on Ag_2_Te films under low-temperature differential conditions. It is expected to be used in wearable devices, sensors, and other applications that convert waste heat from the human body or the environment into electricity to power low-power devices.

## 4. Conclusions

In summary, high-performance n-type Ag_2_Te thin films with dense, uniform, and highly crystalline structures were prepared by using a dual-source co-sputtering method. Precise substrate temperature optimization during magnetron sputtering was performed, and a *PF* of ~3.95 *μ*W cm^−1^K^−2^ at room temperature was achieved for the sample prepared at a substrate temperature of 280 °C. After the further annealing treatment, the carrier mobility and Ag/Te ratios were modified, leading to the enhancement of electrical conductivity, resulting in an improvement in *PF* to ~4.79 *μ*W cm^−1^K^−2^ when annealing at 200 °C for 30 min. Finally, an Ag_2_Te thin-film generator consisting of five legs was constructed, which was able to provide an output voltage of approximately 14.43 mV and a power output of about 50.52 nW at a Δ*T* of 40 K. Ag_2_Te films with good thermoelectric properties have proven to have potential applications in low-power, flexible fields, and wearable electronic devices.

## Data Availability

The original contributions presented in the study are included in the article, further inquiries can be directed to the corresponding author.

## Supplementary Materials

The following supporting information can be downloaded at: https://www.mdpi.com/article/10.3390/nano14211762/s1, Figure S1. (a–c) Temperature-dependent electrical conductivity (*σ*), Seebeck coefficient (S), power factor (PF) at different substrate temperatures. Figure S2. EDS results and element mappings for ST-280 sample. Figure S3. (a–e) Surface morphology of Ag2Te films prepared at different annealing temperatures and of the unannealed sample.
