# Supplementary material for: Optimization of Thermoelectric Performance of Ag2Te Films via a Co-Sputtering Method
Source: Nanomaterials (Basel). 2024 Nov 3;14(21):1762. doi: 10.3390/nano14211762 (PMC11547814; doi:10.3390/nano14211762)
Supplement: Supplementary file 1 [file nanomaterials-14-01762-s001.zip › nanomaterials-3290602-supplementary.pdf]

nanomaterials

Supporting Information

Optimization of Thermoelectric Performance of Ag_2Te Films via a Co-Sputtering Method

Hanwen Xu, Zhongzhao Zha, Fu Li, Guangxing Liang, Jingting Luo, Zhuanghao Zheng and Yue-Xing Chen *

Institute of Thin Film Physics and Applications, Shenzhen Key Laboratory of Advanced Thin Films and Applications, Key Laboratory of Optoelectronic Devices and Systems of Ministry of Education and Guangdong Province, State Key Laboratory of Radio Frequency Heterogeneous Integration, College of Physics and Optoelectronic Engineering, Shenzhen University, Shenzhen 518060, China; 2200451025@email.szu.edu.cn (H.X.); 2300451029@email.szu.edu.cn (Z.Z.); lifu@szu.edu.cn (F.L.); lgx@szu.edu.cn (G.L.); luojt@szu.edu.cn (J.L.); zhengzh@szu.edu.cn (Z.Z.)

* Correspondence: chenyx@szu.edu.cn

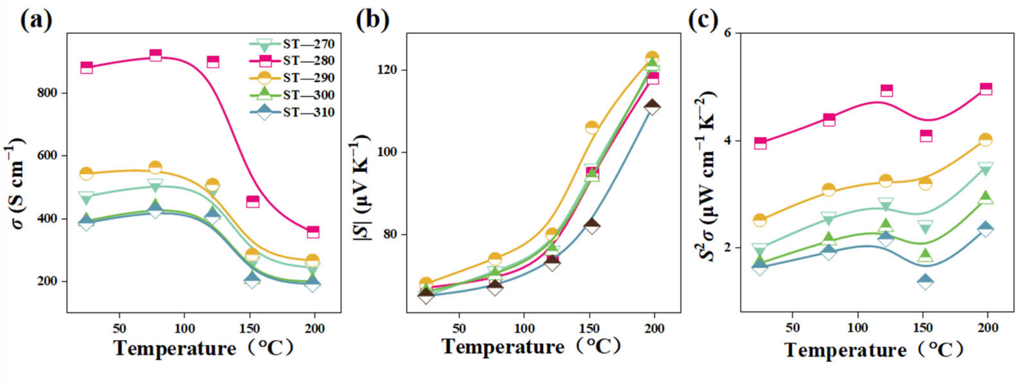

Figure S1. (a–c) Temperature-dependent electrical conductivity (σ), Seebeck coefficient (S), power factor (PF) at different substrate temperatures.

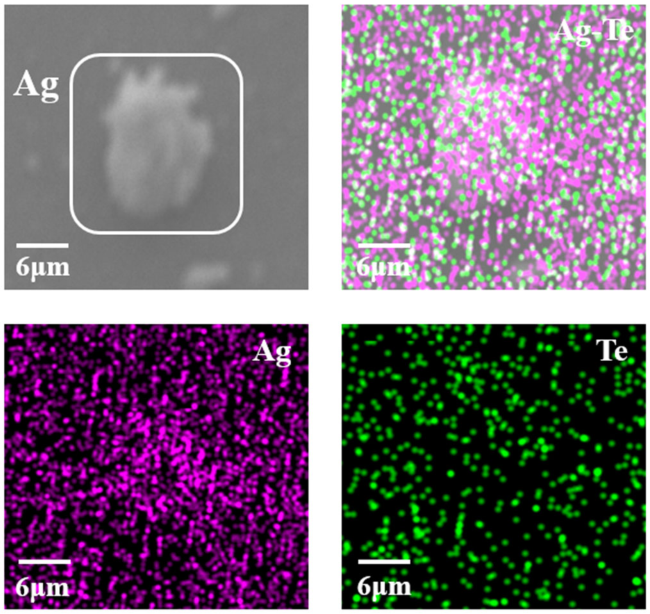

Figure S2. EDS results and element mappings for ST-280 sample.

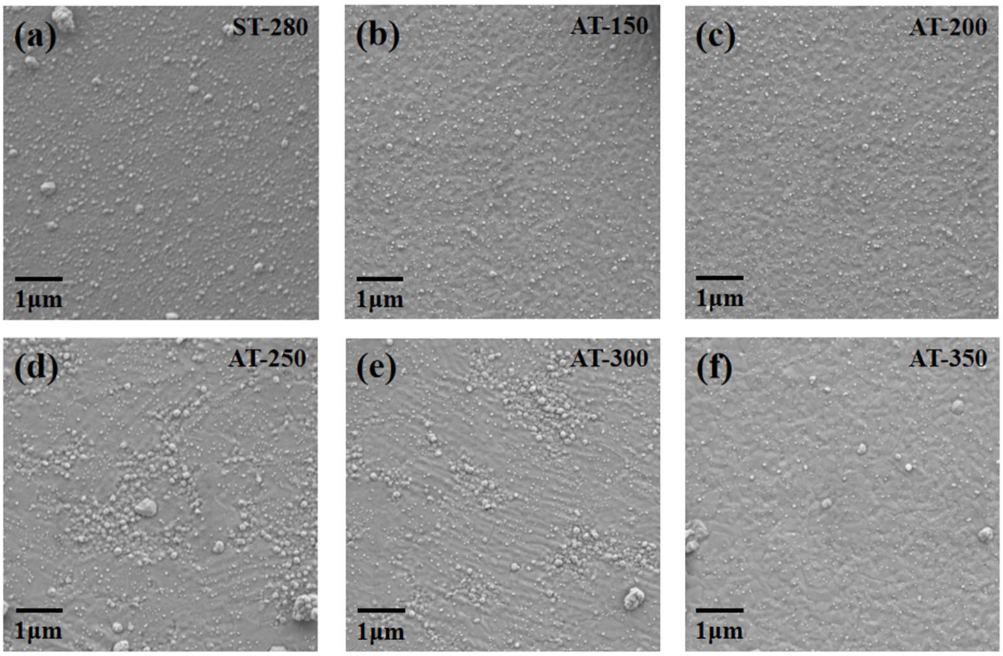

Figure S3. (a–e) Surface morphology of Ag_2Te films prepared at different annealing temperatures and of the unannealed sample.

Disclaimer/Publisher's Note: The statements, opinions and data contained in all publications are solely those of the individual author(s) and contributor(s) and not of MDPI and/or the editor(s). MDPI and/or the editor(s) disclaim responsibility for any injury to people or property resulting from any ideas, methods, instructions or products referred to in the content.
